# Patient-Specific Mandibular Reconstruction Plates Increase Accuracy and Long-Term Stability in Immediate Alloplastic Reconstruction of Segmental Mandibular Defects

**DOI:** 10.1007/s12663-019-01323-9

**Published:** 2020-01-03

**Authors:** A. N. Zeller, M. T. Neuhaus, L. V. M. Weissbach, M. Rana, A. Dhawan, F. M. Eckstein, N. C. Gellrich, R. M. Zimmerer

**Affiliations:** 1 Department of Oral and Maxillofacial Surgery, Hannover Medical School, Hannover, Germany; 2 Department of Oral and Maxillofacial Surgery, University Hospital Duesseldorf, Düsseldorf, Germany; 3 Sri Guru Ram Das Institute of Dental Sciences and Research, Amritsar, India

**Keywords:** Ablative surgery, Alloplastic reconstruction, CAD/CAM, Mandibular reconstruction, Fracture, Osteosynthesis

## Abstract

**Objectives:**

The aim of the current study was to evaluate potential differences in the accuracy of mandibular reconstruction and long-term stability, with respect to different reconstructive procedures.

**Methods:**

In total, 42 patients who had undergone primary segmental mandibular resection with immediate alloplastic reconstruction, with either manually pre-bent or patient-specific mandibular reconstruction plates (PSMRP), were included in this study. Mandibular dimensions, in terms of six clinically relevant distances (capitulum [most lateral points], capitulum [most medial points], incisura [most caudal points], mandibular foramina, coronoid process [most cranial points], dorsal tip of the mandible closest to the gonion point) determined from tomographic images, were compared prior to, and after surgery.

**Results:**

Dimensional alterations were significantly more often found when conventionally bent titanium reconstruction plates were used. These occurred in the area of the coronoid process (*p* = 0.014). Plate fractures were significantly (*p* = 0.022) more often found within the manually pre-bent group than within the PSMRP group (17%/0%).

**Conclusion:**

The results suggest that the use of PSMRP may prevent rotation of the proximal mandibular segment, thus avoiding functional impairment. In addition, the use of PSMRP may potentially enhance the long-term stability of alloplastic reconstructions.

## Introduction

Alloplastic reconstruction of the mandible remains as one of the most challenging procedures in craniomaxillofacial surgery. After ablative surgery, the aim of the reconstructive procedure is long-term stability and full oral rehabilitation, as well as the preservation of the facial esthetics of the patient [1–3]. Complex interactions between bony dimensions, joint function, and muscular interactions make accurate reconstruction necessary. In analogy to the aims of orthognathic surgery, some authors have suggested that it is essential to achieve a centric condyle position [4], even though there is no uniform definition of this term, nor sufficient data to support its use. Furthermore, iatrogenic discrepancies in postsurgical mandibular dimensions have been proposed to trigger neuromuscular dysfunctions such as cranio-mandibular dysfunction (CMD) [5, 6].

The first surgical attempts of mandibular reconstruction date back to the seventeenth to nineteenth centuries [7]. Initially performed without rigid internal fixation, osteosynthesis plates were first introduced in the twentieth century. Traditionally, stock plates had to be intraoperatively adapted to the anatomy of each patient. Plate bending is suspected to be the major cause for delayed fractures of implanted material [8]. As this is not necessary for patient-specific, computer-aided designed and computer-aided manufactured (CAD/CAM) plates, they can be made from stiffer and more durable materials [9] not suitable for manual bending. In addition to this, screw holes can be added selectively, and only in areas where surgically required. While fractures are relatively rare, there have been increasing reports of such complications in recent years [10].

The CAD/CAM method has become increasingly used, especially in routine surgical procedures [11–15]. For reconstructive procedures, the first step involves the initial pre-bending of standard reconstruction plates, prior to surgery, using stereolithographic biomodels of the mandible [11, 16]. This preparatory step serves to reduce the operating time required. Milled reconstruction plates and implants have also been introduced for use in reconstructive procedures. Milling in general is associated with design limitations, especially in terms of functionalized elements, such as integrated positioning aids or extensions for primary dental rehabilitation. Additively manufactured implants [12] have been able to overcome these limitations. Some studies have suggested beneficial effects of patient-specific CAD/CAM implants [17], as well as positioning devices [18, 19], on operating times and surgical outcomes. Yet, very few studies have investigated the effects of patient-specific implants on outcomes following mandibular reconstruction [9, 20]. Nevertheless, patient-specific mandibular reconstruction plates (PSMRP) and pre-bent plates have almost fully displaced conventional stock osteosynthesis plates in some institutions, as reports have shown significantly reduced operating times (by 0.4–1.4 h) for mandibular reconstructive procedures [21, 22]. At present, the general benefits and disadvantages of patient-specific mandibular reconstruction, in terms of treatment outcomes, remain unclear.

Therefore, the aim of the current study was to evaluate the accuracy and long-term stability of mandibular reconstruction using pre-bent stock reconstruction and PSMRP.

## Materials and Methods

### Patient Inclusion and Exclusion Criteria

This retrospective, monocentric study was conducted at the Department of Cranio-Maxillo-Facial Surgery at Hannover Medical School and approved by the institutional review board (Approval number: 2281-2014).

The department’s database was screened for patients with segmental defects who had undergone primary mandibular resection and immediate alloplastic reconstruction with different reconstruction plates between 2013 and 2017. Pre- and postoperative patient records, as well as intraoperative documents, were analyzed.

Inclusion criteria were patients with primary segmental mandibular defects, including the midline, with immediate alloplastic reconstruction using either pre-bent plates or PSMRP. Defects including either condylar or coronoid processes, as well as immediate bony reconstructions, were excluded. In addition, secondary alloplastic reconstructions for preexisting segmental defects with fractured plates were also excluded. Cases without pre- and postoperative 3D imaging [i.e., computed tomography (CT) or cone beam computed tomography (CBCT)], or having major metal or motion artifacts in tomographic imaging, were not eligible for inclusion.

### Pre-bent Plates and PSMRP

**Fig. 1:**
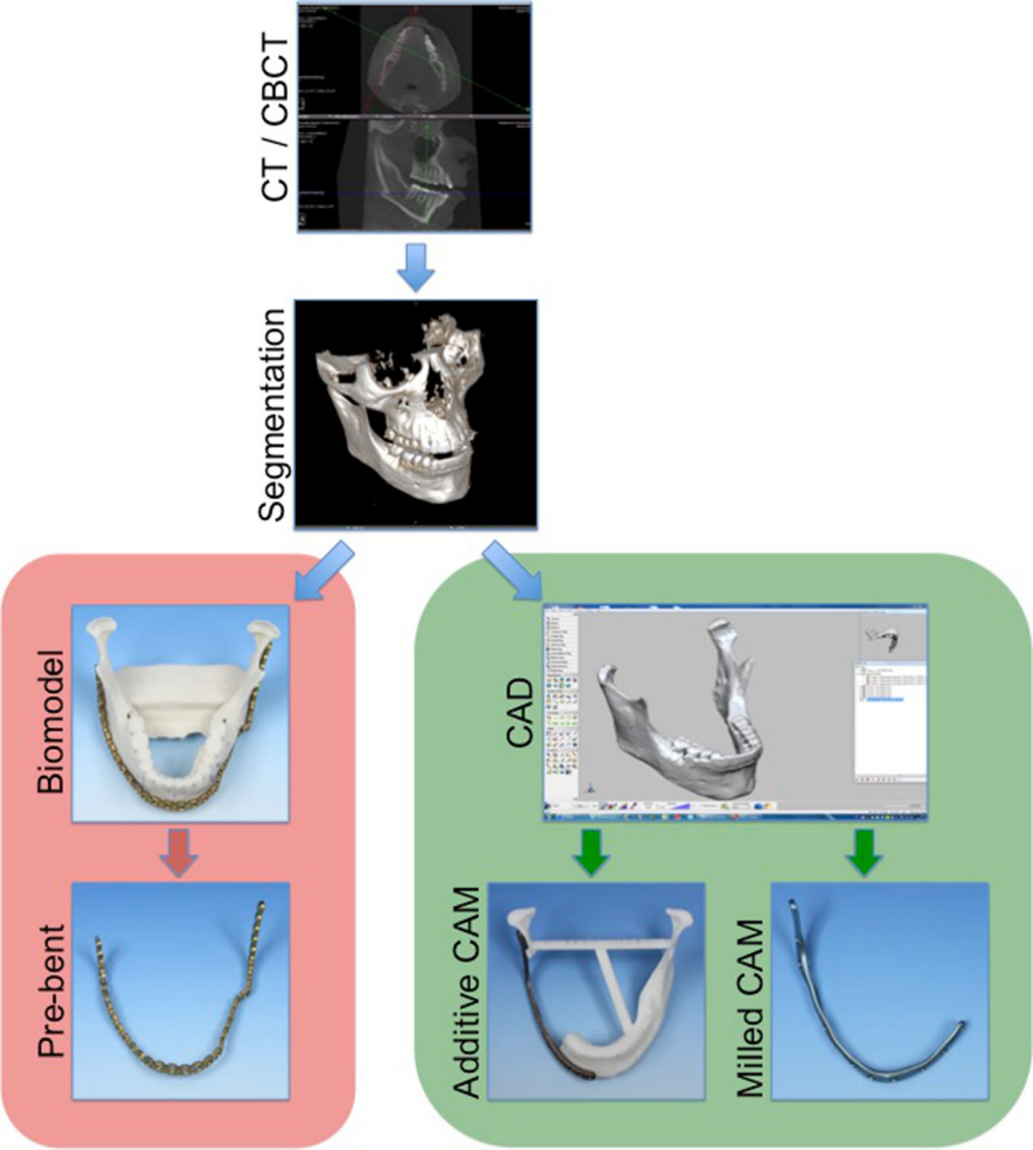
Workflow for manually pre-bent (red) and CAD/CAM reconstruction plates (green)

Both methods were based on data acquired by CT or CBCT (Fig. 1). For each case, Digital Imaging and Communications in Medicine (DICOM) data were imported into a surgical planning software (iPlan CMF Version 3.0 Brainlab, Feldkirchen, Germany). Volumetric models were generated by a standardized combination of threshold-based and atlas-based segmentation. Models were exported as “.stl” data. In the case of manual pre-bending, biomodels were digitally reinforced with a bar connecting both mandibular angles from the inside to avoid fractures and were subsequently printed from resin-bonded plaster (Phacon, Leipzig, Germany). Standard 2.8 mm titanium reconstruction plates (Synthes, Zuchwil, Switzerland) were contoured onto these models by manual bending prior to surgery and then sterilized according to the manufacturer’s specifications.

For PSMRP, DICOM data were transferred to assign industrial partners for virtual planning and manufacturing (KLS-Martin, Tuttlingen, Germany and Depuy Synthes, West Chester, USA). Plates were produced, depending on the manufacturer’s protocol, either by milling from titanium blocks (CMF Trumatch©, Depuy Synthes, West Chester, USA) or additively from a titanium–aluminum alloy powder (selective laser melting (SLS), KLS-Martin, Tuttlingen, Germany) according to the department’s standard designs.

## 3D Image Data Analysis

**Fig. 2:**
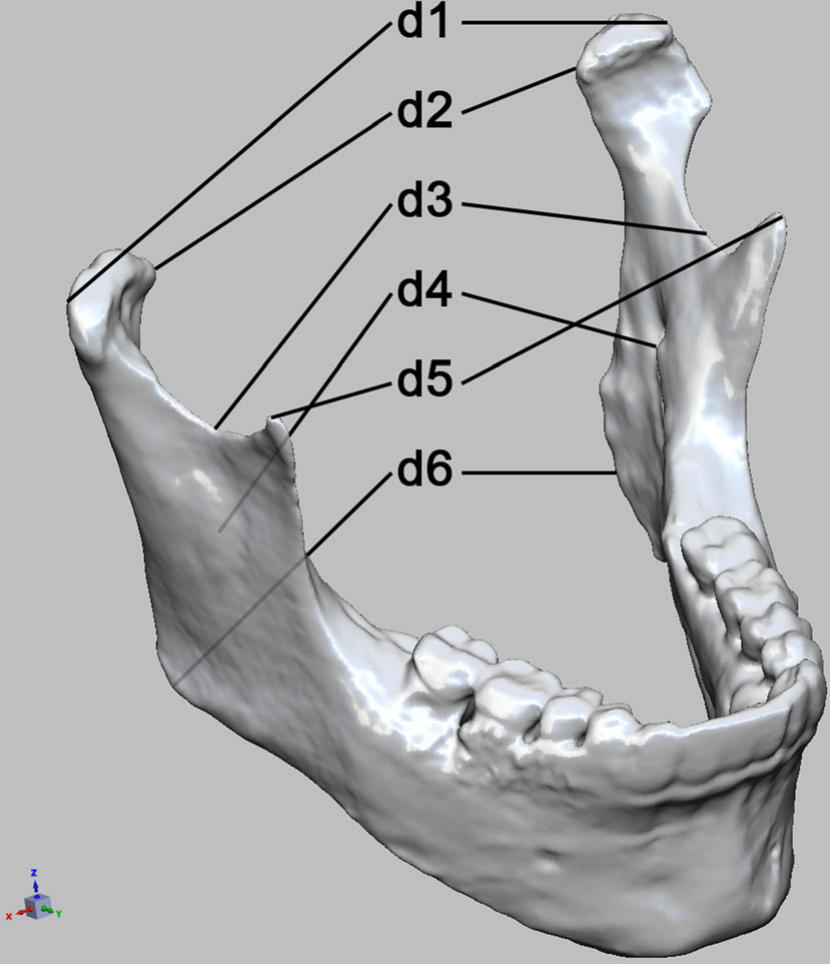
Measured distances prior to, and after alloplastic reconstruction

**Fig. 3:**
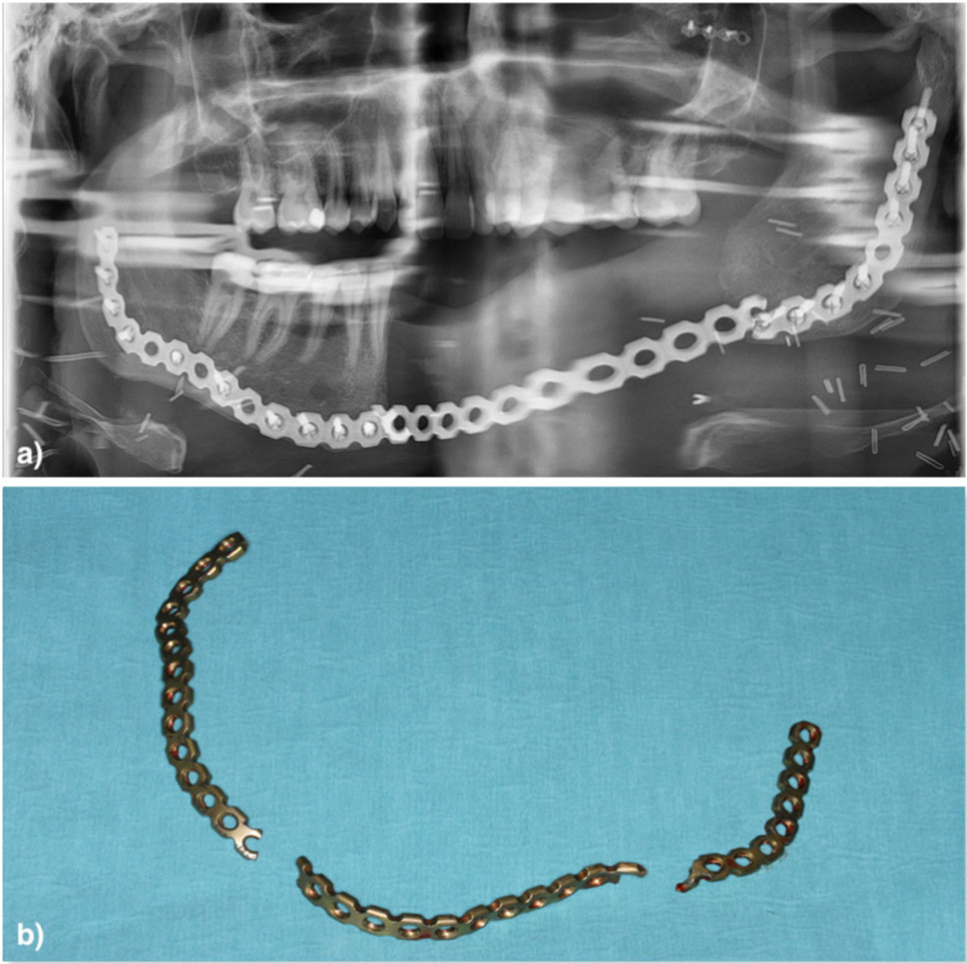
**a** Panoramic X-ray of a fractured reconstruction plate, **b** clinical view after fracture

Six clinically relevant distances modified after Wilde et al. [19, 23] were manually measured in both pre- and postoperative images after three-dimensional alignment in a DICOM viewer (Visage 7^®^, Visage Imaging, Inc.) (Fig. 2, Table 1). All alignments and measurements were repeated three times in total. Additionally, all patients were followed up until mid-2018, and fractures (Fig. 3) of reconstruction plates were recorded.

**Table 1:** Description of distances measured

d1	Capitulum—most lateral points (l/r)
d2	Capitulum—most medial points (l/r)
d3	Incisura—most caudal points (l/r)
d4	Mandibular foramina (l/r)
d5	Coronoid process—most cranial points (l/r)
d6	Dorsal tip of the mandible closest to Go point (l/r)

Distances “d” between corresponding mandibular landmarks of the left (l) and right (r) side

### Statistics

Measured distances (indicated as “d” in Fig. 2.) were collected in Microsoft Excel for Mac 14.7.7 (Microsoft Corp. Redmond, USA). After calculating mean values and their relative differences in pre- and postoperative distances (as a quotient, indicated as “Q”) and their absolute value, data were processed with Wizard^®^ Version 1.9.16 by Evan Miller. The Shapiro–Wilk test was used to evaluate the data distribution. The Mann–Whitney test was performed to compare changes in distance between the pre-bent plate and PSMRP groups. A *p* value of < 0.05 was considered to be significant. Further statistical analyses and verification of obtained results were carried out using IBM SPSS Statistics 24^®^ (IBM Corp. Armonk, NY, USA).

## Results

### Patient Follow-Up

Forty-two patients met the inclusion criteria, with 12 (28.6%) having undergone mandibular reconstruction with pre-bent stock osteosynthesis plates. Patient-specific implants were used for 30 patients, of whom 15 (35.7%) received additively manufactured plates and 15 (35.7%) received milled titanium plates for mandibular continuity defects. Thus, a total of 30 (71.4%) of the evaluated cases were reconstructed using CAD/CAM osteosynthesis. The average time elapsed between pre- and postoperative imaging was 5.5 ± 1.74 months, and the average clinical follow-up time was 37.67 months.

### Accuracy of Mandibular Reconstruction

Relative changes in the six assessed distances, apart from Q1, Q3, and Q4, were found to be not normally distributed (Shapiro–Wilk test). Therefore, the Mann–Whitney test was used for statistical comparisons.

**Fig. 4:**
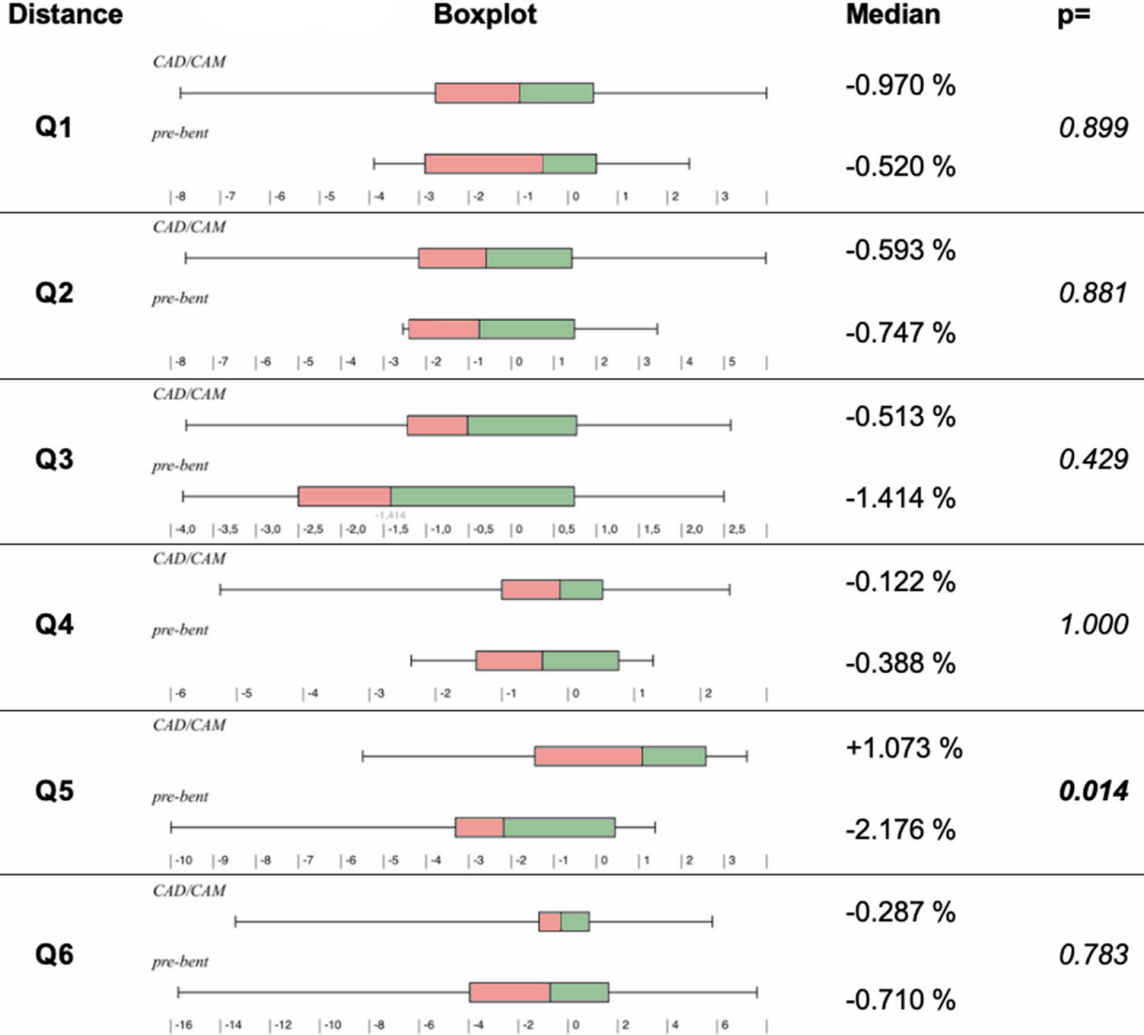
Comparison of postoperative changes in mandibular dimensions between the pre-bent plate group and PSMRP group

A general trend for an increase in dimensional changes was observed, caudally to cranially (Fig. 4). Changes in distances assessed in the area of the coronoid process (d5) were significantly different between pre-bent and PSMRP groups (+1.073% vs. − 2.176% by median, respectively, *p* = 0.014). Differences in Q1-4 and Q6 were not statistically significant between the groups. Regarding absolute differences, no statistical significance was found.

### Fractures

**Fig. 5:**
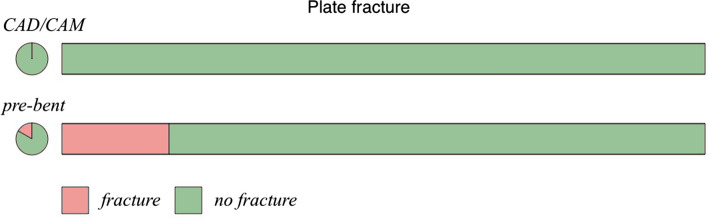
Reconstruction plate fractures according to plate type

The average follow-up time was 52.25 months for the pre-bent group, and 31.83 months for the PSMRP group (Fig. 5). Fractures only occurred within the pre-bent group (2 out of 12, *p* < 0.022).

## Discussion

Postoperative changes in mandibular dimensions have been suspected to be a cause of functional disorders such as CMD [4, 6]. Major differences may even cause physical obstruction of muscular and bony structures crucial for orofacial function, such as the temporal muscle. Thus, accuracy of reconstruction is one of the major aims of reconstructive procedures after ablative surgery of the mandible. A large variety of instruments, ranging from custom drill guides [19] and digital planning algorithms [24], to patient-specific implants are available to the surgeon. It has been postulated that these measures facilitate an increased accuracy [19] and reduction in intra-surgical time required [16].

The current study shows that the accuracy of mandibular reconstruction in some areas can be increased by the use of digitally planned, individual CAD/CAM reconstruction plates. Conventional procedures were associated with an increasing loss of accuracy in the caudo-cranial direction. Procedures involving patient-specific implants showed significantly less postoperative compression in the area of the coronoid process. This suggests that patient-specific implants are better able to preserve preoperative mandibular dimensions, mainly by preventing rotation of the proximal mandibular segments. This is especially important when two-stage procedures for bony reconstruction are planned for the long-term durability of alloplastic reconstruction. As documented in this study, conventionally bent osteosynthesis plates also fracture more often. As adaptation to existing mandibular dimensions often requires reciprocal bending, the increased susceptibility of conventionally bent plates to fractures may be expected, given the stresses induced by cold bending. Indeed, prior studies have shown that strongly angulated areas have the highest rates of fracture [25].

CAD also provides the possibility of finite element analysis before manufacturing [26]. Areas subject to increased mechanical stress can be redesigned before manufacturing to improve medium and long-term implant stability. This may explain the lack of fractures in the PSMRP group, although potential bias is acknowledged due to the follow-up period being longer for the pre-bent plate group. As intraoperative corrections of positioning, in terms of implant geometry, are not possible when CAM plates are used, special attention must be given to the CAD process. Apart from the surgeon’s skills, a number of factors, including segmentation errors, artifacts, and tolerances in implant and bio-model manufacturing, are crucial for the stability of postoperative mandibular dimensions. The results of this study also suggested a decrease in mandibular width when using pre-bent plates. This may have been due to cumulative errors in model segmentation, model printing, and inaccuracies in manual bending.

## Conclusion

Prior to alloplastic mandibular reconstruction, surgeons have the choice of conventional, pre-bent, or CAD/CAM plates. Each of these has their advantages and disadvantages. These should be considered carefully on a case-by-case basis. The current study clearly shows the superior stability of CAD/CAM plates compared to manually bent plates. Furthermore, implantation of pre-bent conventional plates was associated with comparably higher aberrations in mandibular dimensions in some areas. The results of this study suggest that cases of mandibular reconstruction requiring long-term stability of the alloplastic material should consider the use of CAD/CAM plates. In cases where postoperative mandibular dimensions may not be preserved by the patient’s occlusion, CAD/CAM plates may also be a means of avoiding proximal segment rotation.
